# Influenza Vaccination Coverage Among Elderly Patients with Chronic Lung Respiratory Disease in Ningbo, China: Impact of Free Vaccination Policies and the COVID-19 Pandemic

**DOI:** 10.3390/vaccines13070705

**Published:** 2025-06-29

**Authors:** Xiaoqing Wu, Jieping Chen, Pingping Li, Tianchi Yang, Lixia Ye

**Affiliations:** 1 School of Public Health, Ningbo University, Ningbo 315211, China; 15082864833@163.com; 2 Ningbo Municipal Center for Disease Prevention and Control, Ningbo 315010, China; bentley1982@163.com; 3 Jiangbei District Center for Disease Prevention and Control, Ningbo 315021, China; lipingping0218@163.com

**Keywords:** influenza vaccine, coverage, COVID-19, free vaccination policy, determinants, chronic lower respiratory diseases

## Abstract

Background: Elderly patients with chronic lower respiratory diseases (CLRDs) demonstrate an increased susceptibility to complications arising from influenza. Influenza vaccination remains the most effective strategy against influenza-related diseases among elderly CLRD patients. This study aimed to evaluate the influenza vaccination status of older CLRD patients and the factors affecting influenza vaccination. Methods: Using population-based health registries, we analyzed the longitudinal uptake of influenza vaccination among elderly patients with CLRDs in Ningbo from the 2018/19 season to the 2022/23 season. A multivariate logistic regression analysis was performed to identify behavioral determinants influencing influenza vaccination among elderly CLRD patients under Ningbo’s post-pandemic free vaccination policy. Results: An average of 487,309 older patients with CLRDs were included in our analysis for each season. The influenza vaccination rate increased from 3.59% in 2018/19 to 43.32% in the 2022/23 influenza season. There was a significant increase in the proportion of timely influenza vaccinations prior to 15 November, rising from 3.01% before the COVID-19 pandemic to 33.90% during the pandemic period. The multivariate logistic regression analysis indicated that both the COVID-19 pandemic and free vaccination policy significantly promoted influenza vaccine uptake. Older CLRD patients with comorbidities such as diabetes, hypertension, or cancer exhibited higher influenza vaccination coverage, whereas those who have experienced acute cardiovascular events showed a lower vaccination rate. Additionally, a prior vaccination history significantly influenced uptake. Conclusions: Despite the significant improvement in vaccination rates, coverage among elderly patients with CLRDs remains below the WHO target. Addressing this gap requires integrated interventions that combine expanding the population eligible for free vaccinations, community mobilization efforts, and effective communication regarding cardiovascular safety to mitigate vaccine hesitancy within high-risk groups.

## 1. Introduction

Chronic lower respiratory diseases (CLRDs) in elderly patients significantly increase vulnerability to influenza infection, exacerbating existing pulmonary conditions and worsening the clinical prognosis in this population [1]. There is evidence [2] that indicates that older adults with chronic comorbidities experience 1.8-fold higher hospitalization rates during influenza seasons compared to their healthy counterparts, with 72% of influenza-related in-hospital deaths occurring in this high-risk group. The synergistic impact of CLRDs and influenza infection has emerged as a critical public health concern, imposing substantial health and socioeconomic burdens on healthcare systems.

Influenza vaccination remains the cornerstone of preventive strategies against influenza-related morbidity in vulnerable populations, such as CLRD patients [3]. For elderly CLRD patients, immunization confers significant protection against respiratory complications, demonstrating a 55% risk reduction in laboratory-confirmed influenza among asthmatics [4] and a 22% decrease in COPD-related hospitalizations [5,6]. Despite World Health Organization (WHO) recommendations for annual vaccination for chronic disease groups [7], the influenza vaccination coverage among elderly CLRD patients varies worldwide: 37.3% in South Korea [8], 65.02% in Spain [9], and 75.0% in France [10]. The exclusion of influenza vaccines from China’s National Immunization Program has created systemic barriers to vaccine accessibility, leading to lower vaccination coverage in high-risk populations.

In response to the COVID-19 pandemic, a free influenza vaccination program was initiated in Zhejiang province, which aimed to increase influenza vaccination rates among older adults. In 2020, individuals aged 70 years and older in Zhejiang were eligible for the free trivalent inactivated influenza vaccine. In 2021, this eligibility was expanded to those aged 65 years and above. Several studies [8,11] have indicated that the COVID-19 pandemic and the implementation of a free vaccination policy significantly influenced influenza vaccination rates. While preliminary studies suggest that policy interventions significantly influence vaccination behaviors [12], the extant evidence remains constrained by pre-pandemic data collection and geographically limited implementation scopes. No contemporary studies have systematically evaluated vaccination patterns among elderly CLRD patients under China’s evolving immunization landscape.

The study analyzed longitudinal data (2018/19–2022/23) from Ningbo’s health information platforms to delineate the temporal vaccination trends among elderly CLRD patients and identify the determinants of vaccine uptake within the evolving post-pandemic policy landscape.

## 2. Materials and Methods

### 2.1. Data Resources

This study utilized two health information systems in Ningbo: the Ningbo Regional Health Information Platform (NRHIP) and the Zhejiang Immunization Information Management System (ZJIIMS). As a national pioneer in digital health infrastructure, Ningbo’s NRHIP systematically consolidates electronic medical records (EMRs) from all 65 public hospitals and 154 primary care institutions within its jurisdiction, ensuring citywide population coverage of clinical data. The ZJIIMS, a provincial-level immunization registry, maintains comprehensive vaccination histories for Zhejiang residents. We extracted de-identified patient records up to 30 June 2023 using China’s Unique National Identification Numbers (NIDs), enabling cross-system integration of medical histories, immunization status, and sociodemographic profiles. In addition, the mortality data for the study population was updated in accordance with the data from the Ningbo Death Surveillance Information System using the Unique NIDs. 

### 2.2. Study Population

This study employed a dynamic eligibility framework aligned with China’s influenza vaccination schedule, defining observational periods from 1 July to 30 June of the next year as annual cycles. Participants were included in the study if they met the following criteria during any influenza season between 2018/19 and 2022/23: (1) born prior to 31 December 1962; (2) registered in the ZJIIMS and residing in Ningbo throughout the study period; (3) alive at the commencement of each influenza season; and (4) diagnosed with a Chronic Lower Respiratory Disease (CLRD) at the commencement of each influenza season. The records for CLRD diagnosis were obtained from the NRHIP based on the codes J40–J47 from the International Classification of Diseases, Tenth Revision (ICD-10).

### 2.3. Measures

We conducted a retrospective observational study, analyzing influenza vaccine uptake among older adults with CLRDs across different influenza seasons. The primary outcome, vaccine coverage rate (VCR), was operationally defined as the proportion of vaccinated individuals relative to the eligible cohort in each influenza season.

The free influenza vaccination program was launched in September 2020 in Ningbo. During the 2020/21 season, the program targeted permanent residents aged 70 years and older. In the subsequent influenza season (2021/22), the target population was expanded to individuals aged 65 years and above. In this study, permanent residents born before 31 December 1950 were eligible to receive the influenza vaccine at no cost during the 2020/21 season. Those born prior to 31 December 1956 qualified for free influenza vaccination in the 2021/22 season, while individuals born before 31 December 1957 were entitled to complimentary influenza vaccination in the 2022/23 season. Based on these criteria, five cohorts were selected as our target groups to assess the impact of the free vaccination policy on influenza vaccine uptake (Table 1).

Based on the fact that the COVID-19 pandemic began in January 2020 (the influenza vaccination for the 2019/20 season was nearly completed by this time), the study periods were divided into two distinct phases: the pre-COVID-19 pandemic period (2018/19–2019/20) and the COVID-19 pandemic period (2020/21–2022/23).

Our study incorporated three principal covariate domains: demographic characteristics, comorbidity profiles, and vaccination histories. Demographic data (gender, date of birth, immigration status, and residential classification) were extracted from the ZJIIMS. Residential status was stratified into permanent residents (individuals with registered household and physical residency in Ningbo) and migrants, while urban–rural designation followed municipal administrative divisions: Haishu, Jiangbei, Beilun, Zhenhai, Yinzhou, and Fenghua districts as urban areas and Xiangshan, Ninghai, Yuyao, and Cixi as rural regions. Comorbidity data, including influenza-like illness (ILI) diagnoses from the preceding season and chronic conditions (diabetes, hypertension, cancer, stroke, acute myocardial infarction, and heart failure) that have been clinically validated to influence vaccination behavior, were retrieved from the Ningbo Regional Health Information Platform (NRHIP) using ICD-10 codes, with all diagnoses temporally constrained to before 1 July of each influenza season (Supplementary Table S1). Vaccination history records were retrieved for three types of immunization: (1) seasonal influenza vaccines in last influenza season, (2) COVID-19 vaccines, and (3) 23-valent pneumococcal polysaccharide vaccines (PPSV23), which were verified through the ZJIIMS with an acquisition cutoff date of 1 July for each influenza seasons.

### 2.4. Statistical Analysis

We performed a descriptive analysis on all variables, incorporating counts and proportions. The influenza vaccination rates among the study population across various subgroups were calculated. Associations between influenza vaccine uptake and demographic factors, vaccination policies, comorbidities, and vaccination history were evaluated using multivariate logistic regression. Adjusted factors included demographic characteristics, eligibility for free influenza vaccination, a diagnosis of ILI in the previous influenza season, and prior vaccination history. Adjusted odds ratios (aORs) along with 95% confidence intervals (95% CIs) are presented. Two-tailed *p*-values < 0.05 were deemed statistically significant. The analysis was performed using STATA version 17 for Windows (StataCorp LLC, College Station, TX, USA). The figure was generated using Microsoft Excel.

## 3. Results

### 3.1. Description of the Study Population

The study population consisted of an average of 485,749 older adults with CLRDs across five consecutive influenza seasons (2018–2019 to 2022–2023). The cohort exhibited a balanced gender distribution, with females comprising 50.8–50.9% and males accounting for 49.1–49.2%. A notable predominance of urban residency was observed, ranging from 54.24% (343,202/632,751) to 61.29% (170,892/278,814), while the age group of 70–79 years represented the largest stratum at 35.76–36.44%. Over 94% of participants were permanent residents, whereas migrants constituted approximately 4.22–5.24%. The comorbidity profiles indicated that the prevalence of hypertension (approximately 40%) exceeded that of diabetes (11.78–12.45%), and between 3.02% and 7.97% had a history of acute cardiovascular events. The vaccination history included influenza vaccination during the previous season (which ranged from 2.48% to 36.73%) as well as PPSV23 vaccination history (0.71–2.29%). Additionally, diagnoses related to influenza-like illnesses varied between seasons, with rates from 30.00% (189,795/632,751) to 81.41% (226,980/278,814). Detailed information is presented in Table 2.

### 3.2. Influenza Vaccination Coverage Among Older Adults with CLRDs

**Figure 1:**
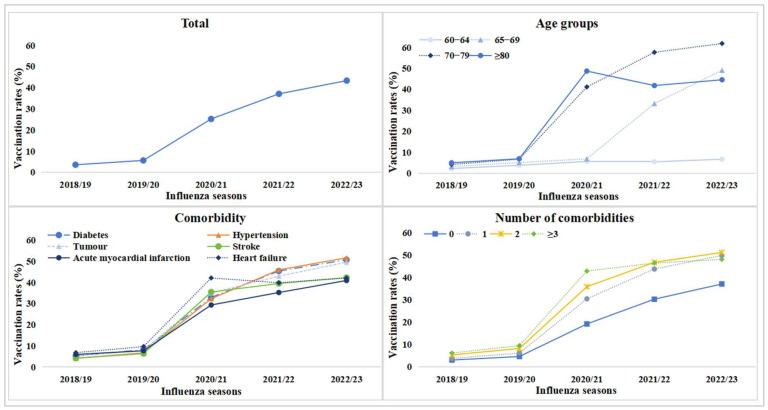
Total influenza vaccination coverage and influenza vaccination coverage by age groups, comorbidity, and number of comorbidities.

The influenza vaccination rates exhibited sustained growth across the study population and major subgroups from 2018/19 to 2022/23 (Figure 1; Supplementary Table S2). Among older adults with CLRDs, the population-wide coverage demonstrated a near 12-fold increase, rising from 3.59% (95% CI: 3.52–3.66%) in 2018/19 to 43.32% (95% CI: 43.20–43.45%) in 2022/23. The most pronounced surge occurred in the 70–79 age cohort, where vaccination rates escalated from 4.13% (4191/101,592) to a remarkable 61.83% (139,898/226,267) over the five-season period. Notably, the comorbidity-stratified analyses revealed consistent annual vaccination increases among older CLRD patients with concurrent chronic conditions, with the exception of heart failure cases. A persistent disparity across all seasons demonstrated that adults with comorbidities maintained significantly higher influenza vaccination rates than those without such diagnoses.

### 3.3. Impact of Free Influenza Vaccination Policy on the Influenza Vaccine Uptake Among Elderly CLRD Patients

**Figure 2:**
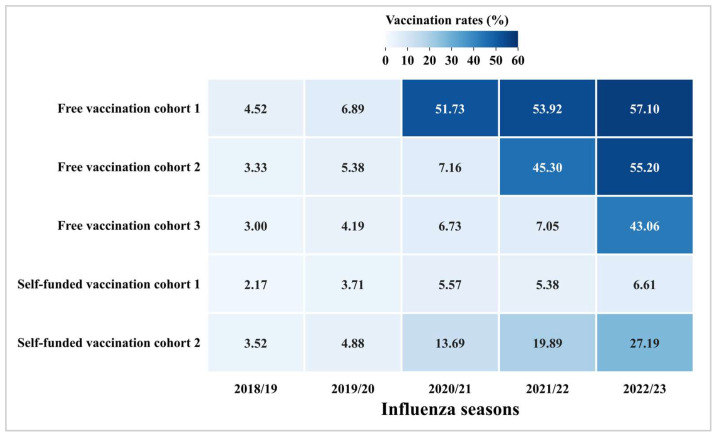
Influenza vaccination coverage in five cohorts from 2018/19 to 2022/23.

To assess the impact of the free influenza vaccination policy, we analyzed coverage rates across five target cohorts (Figure 2). Free vaccination cohort 1 (eligible for free vaccination since 2020/21) demonstrated a marked surge in vaccination coverage from 6.89% (12,360/179,291) to 51.73% (108,126/209,016) during the initial implementation, followed by gradual increases to 53.92% (127,103/235,711) (2021/22) and 57.10% (142,570/249,689) (2022/23). Free vaccination cohort 2 (eligible starting 2021/22) showed comparable growth from 7.16% (10,537/147,267) to 45.30% (76,164/168,149) in the first policy year, rising further to 55.20% (101,396/183,700) in 2022/23. Free vaccination cohort 3 (eligible starting 2022/23) exhibited a similar pattern with coverage increasing from 7.05% (2271/32,194) to 43.06% (15,169/35,224) post-implementation. Conversely, the vaccination coverage rates of the self-funded vaccination cohort 1 did not demonstrate any notable enhancement over the five influenza seasons, with the highest coverage rate standing at only 6.61% (9516/143,980). Although the vaccination coverage of the self-funded vaccination cohort 2 has presented a consistent annual increase since the 2020/21 season, it is noteworthy that the highest vaccination rate did not exceed 30%. Furthermore, the results of logistic regression analysis indicated that the free influenza vaccination policy has had a significant impact on influenza vaccine uptake, especially in the initial phase of implementing the free vaccination policy (2020/21: aOR = 14.60, 95% CI: 14.10–15.12) (Table 3).

### 3.4. Impact of COVID-19 Pandemic on the Influenza Vaccine Uptake Among the Elderly with CLRDs

**Figure 3:**
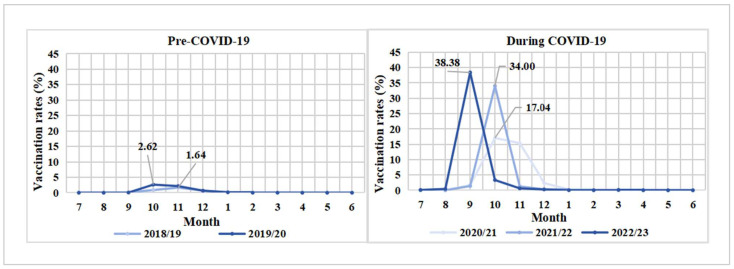
Monthly influenza vaccination coverage by influenza season.

**Figure 4:**
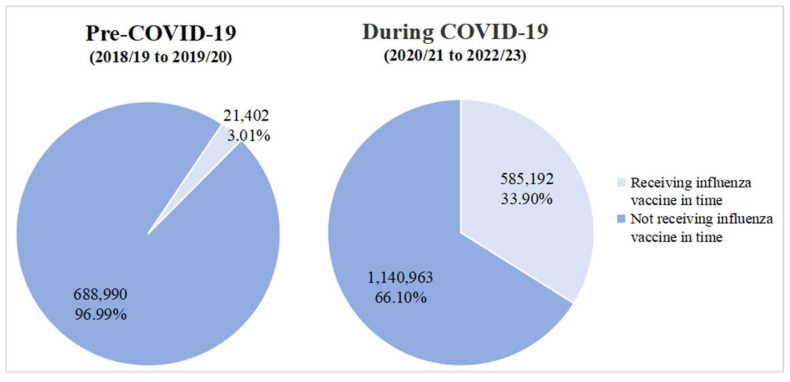
Timely influenza vaccination coverage among older patients with CLRDs before and during the COVID-19 pandemic.

The monthly influenza vaccination coverage across multiple influenza seasons is illustrated in Figure 3. The peak monthly vaccination rate reached 38.38% (242,878/632,751) in September of the 2022/23 season (during the COVID-19 pandemic), while the highest monthly vaccination rate was only 1.64% (4563/278,814) in the 2018/19 season (before the COVID-19 pandemic). The logistic regression analysis also indicated that there was an extremely significant positive correlation between influenza vaccination coverage and the COVID-19 pandemic at the beginning of the COVID-19 pandemic (aOR = 12.71, 95% CI: 12.51–12.91). However, this positive correlation gradually decreased during the following two influenza seasons (2021/22: aOR = 7.31, 95% CI: 7.19–7.42; 2022/23: aOR = 3.98, 95% CI: 3.90–4.06) (Table 3). The timely influenza vaccination rates (the proportion of vaccinated individuals prior to 15 November in each influenza season) among older adults with CLRDs is presented in Figure 4. The prompt vaccination coverage was only 3.01% (21,402/710,392) in the pre-COVID-19 period, while this proportion reached 33.90% (585,192/1,726,963) during the COVID-19 pandemic.

### 3.5. Impact of Comorbidity and Vaccination History on Influenza Vaccine Uptake Among Older CLRD Patients

Our analysis revealed distinct comorbidity-related patterns in influenza vaccination uptake (Table 3). Chronic conditions demonstrated positive associations: diabetes showed an aOR of 1.23 (95% CI: 1.22–1.25), hypertension showed an aOR of 1.32 (95% CI: 1.31–1.33), and cancer showed an aOR of 1.19 (95% CI: 1.16–1.22). Conversely, acute cardiovascular events showed a reduced likelihood: stroke showed an aOR of 0.82 (95% CI: 0.80–0.83), acute myocardial infarction showed an aOR of 0.88 (95% CI: 0.82–0.94), and heart failure showed an aOR of 0.84 (95% CI: 0.82–0.86). In addition, the comorbidity burden demonstrated a significant dose–response relationship, with the CLRD patients with two concurrent conditions showing the highest vaccine uptake (aOR = 1.33, 95% CI: 1.32–1.35) (Table 3).

Vaccination history emerged as a critical determinant of influenza vaccination patterns (Table 3). Prior influenza vaccination demonstrated strong temporal persistence, showing the strongest effect in the pre-COVID-19 vaccination era (2018/19: aOR = 39.41, 95% CI: 37.81–41.08; 2020/21: aOR = 15.63, 95% CI: 15.08–16.20). The PPSV23 recipients maintained a consistent seasonal uptake advantage across all the study periods. A general dose–response gradient was observed across vaccine types (1-dose: aOR = 3.37, 95% CI: 3.27–3.46; ≥2-dose: aOR = 4.23, 95% CI: 3.16–5.66), with COVID-19 vaccination exhibiting threshold-dependent effects in 2022/23—a progressive benefit with ≥2 doses (2-dose: aOR = 1.17, 95% CI: 1.14–1.20; ≥3-dose: aOR = 2.03, 95% CI: 1.98–2.08) versus a reduced likelihood with a single dose (aOR = 0.68, 95% CI: 0.66–0.71).

### 3.6. Association Between Other Factors and Influenza Vaccine Uptake

The associations between demographic characteristics and influenza vaccination rates were slightly different across various seasons (Table 3). Overall, the higher influenza vaccination coverage was observed among women (aOR = 1.15, 95% CI: 1.14–1.16), older adults aged 65 and above (65–69 age group: aOR = 1.21, 95% CI: 1.19–1.23; 70–79 age group: aOR = 1.52, 95% CI: 1.49–1.55; ≥80 age group: aOR = 1.04, 95% CI: 1.01–1.06) and urban residents (aOR = 1.16, 95% CI: 1.16–1.17). The vaccination coverage of local residents was slightly lower than migrants (aOR = 0.48, 95% CI: 0.47–0.49). In addition, the elderly CLRD patients diagnosed with ILIs in last influenza season were more likely to receive the influenza vaccine compared to those without ILIs (aOR = 1.18, 95% CI: 1.17–1.19).

## 4. Discussion

Using population-based health registries in Ningbo, this study examined the dynamics of influenza vaccination among older adults with CLRDs in China over five consecutive influenza seasons, spanning from 2018–2019 to 2022–2023. Our investigation specifically assessed the impact of the COVID-19 pandemic, the implementation of a free influenza vaccination policy, and various clinical determinants, including comorbidity burden, history of ILI, and prior vaccination history, on influenza vaccine uptake patterns within this high-risk respiratory population.

In our study, the average influenza vaccination coverage among older adults with CLRDs reached 22.96% across the five influenza seasons. This indicates that the vaccination coverage for elderly patients with CLRDs was slightly higher than that of the general elderly population, which was reported in our previous study [13] (an average vaccination coverage of 16.59% among the general elderly population over five seasons from 2018/19 264 to 2022/23). Multimorbidity emerged as a significant predictor. The CLRD patients with hypertension, diabetes, or cancer demonstrated a higher vaccination rate. This pattern likely reflects heightened healthcare engagement through frequent medical encounters, coupled with awareness of influenza’s exacerbation risks for chronic respiratory conditions. Paradoxically, acute cardiovascular comorbidities correlated with reduced vaccination rates, potentially attributable to persistent misconceptions regarding vaccine contraindications and amplified safety concerns in cardiopulmonary-compromised individuals. This might also be related to healthcare providers, as some of them are not aware of the clear association between cardiovascular disease and influenza complications. Furthermore, clinicians often prioritize acute-phase interventions for patients with cardiovascular disease, overlooking the long-term benefits of preventive strategies such as vaccination. As a result, they rarely recommend influenza vaccines to these patients. In addition, practical barriers further contribute to insufficient immunization, particularly for stroke survivors facing accessibility challenges in community vaccination settings. Notably, emerging evidence contradicts these precautionary hesitations. The influenza vaccine has demonstrated significant efficacy in the primary and secondary prevention of acute cardiovascular events. A study conducted by Jennifer A Davidson in 2022 [14] demonstrated a 20% acute cardiovascular risk reduction within 28 days post-vaccination in older adults, while Daniel Modin found that mortality benefits increase with cumulative annual immunization [15]. Rigorous safety evaluations have confirmed the safety of influenza vaccines for stabilized cardiovascular patients [16,17,18], reinforcing the current guidelines recommending immunization during clinical quiescence [1].

Our study observed a 14.6-fold increase in influenza vaccination coverage among policy-eligible elderly individuals with CLRDs following the implementation of free vaccination programs, compared to elderly CLRD patients who were not eligible. This finding is consistent with prior evidence regarding the efficacy of subsidized immunization initiatives [8,19,20]. Despite recent reductions in the price of influenza vaccines, pre-2020 costs, particularly for quadrivalent influenza vaccines, imposed disproportionate financial burdens on low-income households. This situation has exacerbated socioeconomic disparities in vaccine uptake [21]. While Ningbo achieved a coverage rate of 43.32% among elderly individuals with CLRDs under the free vaccination policy during the 2022/23 period, this figure remains significantly below the WHO’s target of 75% for populations with chronic diseases. In several high-income countries with long-standing influenza vaccination policies—including Japan, Spain, and South Korea—the coverage rates among elderly individuals have plateaued at moderately high levels [8,22]. Nevertheless, persistent gaps remain when compared to the WHO’s targets and the herd immunity threshold [23]. This suggests that although free vaccination policies effectively promote immunization coverage rates, this single policy instrument remains insufficient to achieve optimal immunization levels. Consequently, multifaceted interventions encompassing targeted public education campaigns should be implemented to mitigate the enduring behavioral and structural obstacles to immunization.

Following the COVID-19 pandemic in 2020, a significant increase in influenza vaccine uptake was observed among elderly CLRD patients in Ningbo, China, with this trend continuing through the subsequent two influenza seasons. Heightened public concern about respiratory diseases, increases in the influenza vaccine supply, and improved vaccination accessibility during the COVID-19 pandemic may have contributed to the sustained rise in vaccination rates. Furthermore, the heightened awareness of respiratory diseases, prompted by public health comparisons between influenza and SARS-CoV-2 regarding symptomatology and transmission, sustained elevated vaccination rates within CLRD populations during the pandemic. This stands in contrast to the investigation by Marco Del Riccio [22], which indicated that influenza vaccination rates among older adults increased in 2020 in most countries (France, Germany, Israel, etc.) but this increase was not sustained in the subsequent two years. This divergence may stem from different COVID-19 pandemic control strategies between countries. China maintained its dynamic zero-COVID policy throughout the pandemic, systematically incorporating influenza vaccination into the epidemic prevention framework to alleviate healthcare system strain. In contrast, most Western countries gradually relaxed restrictions after 2021, leading to diminished public awareness of influenza prevention and consequent declines in vaccination coverage. Considering the 2–4-week period required for antibody development [11], the timing of pre-epidemic seroconversion is crucial for aligning immunization windows with seasonal peaks. Our study demonstrated an increase in timely influenza vaccination coverage during the COVID-19 pandemic, which can be attributed to community-driven mobilization and the strategic deployment of mobile and temporary vaccination units. However, this study reveals that 66.10% of high-risk populations were still unvaccinated prior to 15 November in each influenza season during the COVID-19 pandemic in Ningbo, potentially diminishing immunization effectiveness and increasing healthcare system strain. There is evidence [15,24] to suggest that delayed influenza vaccination is associated with elevated risks of influenza-related infection, hospitalization, and mortality compared to early administration. These findings underscore the need for proactive public health campaigns and targeted behavioral nudges aimed at optimizing adherence to vaccine schedules.

Vaccination history is another significant determinant impacting influenza vaccination. In our study, the older adults with CLRDs who had received the influenza vaccine in the previous season and PPSV23 in the past exhibited superior influenza vaccination coverage. In particular, influenza vaccination history had a strong influence on the influenza vaccination rates before the period of the COVID-19 pandemic. Overall, an increase in the number of doses was correlated with higher influenza vaccination coverage. A similar result was observed in all the people with a COVID-19 vaccination history. This pattern likely reflects CLRD patients’ heightened motivation to mitigate respiratory infection risks that could exacerbate their chronic conditions, coupled with established positive attitudes toward vaccine efficacy. However, there was an exception noted during the 2022/23 influenza season: older adults who had received only one dose of the COVID-19 vaccine exhibited lower rates of influenza vaccination compared to those who had not been vaccinated against COVID-19. This may be due to significant side effects experienced by this population after receiving just one dose of the COVID-19 vaccine, leading to concerns regarding its safety as well as that of other vaccines and resulting in reluctance toward further vaccinations. In fact, serious adverse reactions following the administration of both COVID-19 and influenza vaccines are exceedingly rare [25,26]. One study [27] indicated that elderly patients with COPDs primarily experienced localized reactions such as swelling and lumps post-influenza vaccination; these local reactions typically resolved within one to two days following immunization.

Among the demographic-related factors, female gender, older age, and urban residency were significantly associated with higher influenza vaccination rates, which is consistent with the results of a previous study [13]. It is worth noting that the immigrant population was associated with higher influenza vaccine uptake in our study, which diverges from our previous research [13]. This may be attributed to immigrants’ higher mobility, potentially increasing their exposure risks and consequently strengthening their preventive needs. Meanwhile, Ningbo has made continuous improvements in vaccination services for migrant populations coupled with routine health education to enhance their vaccine awareness and acceptance. However, this also suggests potential deficiencies in local vaccination policy dissemination, as some eligible residents failed to receive an influenza vaccine. These findings underscore the need for targeted interventions to address disparities in vaccine access and improve health communication strategies for both migrant and local populations.

There are several strengths and limitations in our study. The primary strength of this research lies in the extraction of data from large-scale and comprehensive information systems, which allowed for the inclusion of nearly all individuals residing in Ningbo through two platforms, thereby ensuring a sufficient sample size. Additionally, both outcome and covariate variables were sourced directly from the database, effectively eliminating recall bias and enhancing the reliability of the data. However, there are notable limitations to our study. Firstly, despite the correlation between the implementation of the free vaccination policy and rising immunization rates is statistically significant, our pre/post observational study design has inherent limitations in establishing causality due to the absence of a control group. Secondly, it is possible that confounding variables—such as increased public health awareness and concurrent enhancements in healthcare delivery systems—may have also influenced the observed outcomes. Unfortunately, this information was not collected or assessed in our study. Thirdly, it is important to note that our analysis only included data up to 31 June 2023. With the gradual relaxation of COVID-19 prevention and control measures during the post-COVID-19 period, individuals’ vaccination behaviors and willingness may have evolved. Additionally, since September 2024 in Ningbo, individuals aged 60 years and older have become eligible to receive free influenza vaccinations; this change may have led us to underestimate the impact of free vaccination policies on influenza vaccine uptake in our study. Therefore, future analyses should incorporate more recent data and more variables to better reflect the significant effects of both the COVID-19 pandemic and free influenza vaccination policies on vaccination rates among older adults with CLRDs.

## 5. Conclusions

We evaluated the trends in influenza vaccination coverage among elderly CLRD patients from the 2018/19 to 2022/23 influenza seasons in Ningbo, China. The influenza vaccination coverage rose from 3.59% (2018/19) to 43.32% (2022/23). The implementation of free vaccination policies resulted in a 14.6-fold increase in influenza vaccine uptake among policy-eligible populations compared to ineligible groups, which fully demonstrates the significant role of the free vaccination policy in enhancing the vaccination rate. However, in Ningbo, the influenza vaccination coverage among elderly CLRD patients remains below WHO targets and high-income nation benchmarks. Therefore, it is imperative to develop a multimodal strategy that integrates free immunization policies with provider education, community outreach, and evidence-based safety guidance to mitigate the enduring misconceptions about cardiovascular contraindications.

## Figures and Tables

**Table 1:** The base information of the five target cohorts.

Target Group	Date of Birth	Immigration Status	First Season Eligible for Free Vaccination
Free vaccination cohort 1	Before 31 December 1950	Resident	2020/21
Free vaccination cohort 2	1 January 1951–31 December 1956	Resident	2021/22
Free vaccination cohort 3	1 January 1957–31 December 1957	Resident	2022/23
Self-funded vaccination cohort 1	1 January 1958–31 December 1962	Resident/Migrant	/
Self-funded vaccination cohort 2	Before 31 December 1957	Migrant	/

**Table 2:** Characteristics of the study population from 2018/19 to 2022/23 influenza seasons.

Variable	Influenza Seasons				
	2018/19 (N, %)	2019/20 (N, %)	2020/21 (N, %)	2021/22 (N, %)	2022/23 (N, %)
Total	278,814 (100.00)	431,578 (100.00)	510,980 (100.00)	582,424 (100.00)	632,751 (100.00)
Demographic characteristic					
Gender					
Female	141,685 (50.82)	219,863 (50.94)	260,176 (50.92)	296,051 (50.83)	322,306 (50.94)
Male	137,129 (49.18)	211,715 (49.06)	250,804 (49.08)	286,373 (49.17)	310,445 (49.06)
Age groups (years) ^a^					
60–64	57,208 (20.52)	92,745 (21.49)	111,970 (21.91)	129,093 (22.16)	143,980 (22.75)
65–69	73,393 (26.32)	115,642 (26.8)	137,888 (26.99)	157,864 (27.10)	173,509 (27.42)
70–79	101,592 (36.44)	155,486 (36.03)	183,236 (35.86)	209,072 (35.90)	226,267 (35.76)
≥80	46,621 (16.72)	67,705 (15.69)	77,886 (15.24)	86,395 (14.83)	88,995 (14.06)
Region					
Rural	107,922 (38.71)	189,432 (43.89)	230,450 (45.10)	265,934 (45.66)	289,549 (45.76)
Urban	170,892 (61.29)	242,146 (56.11)	280,530 (54.90)	316,490 (54.34)	343,202 (54.24)
Immigration status					
Migrant	11,763 (4.22)	19,098 (4.43)	23,738 (4.65)	28,212 (4.84)	33,141 (5.24)
Resident	267,051 (95.78)	412,480 (95.57)	487,242 (95.35)	554,212 (95.16)	599,610 (94.76)
Eligible for free influenza vaccination	0 (0.00)	0 (0.00)	209,016 (40.90)	403,860 (69.34)	468,613 (74.06)
Diagnosed with ILI last influenza season	226,980 (81.41)	316,319 (73.29)	262,820 (51.43)	233,836 (40.15)	189,795 (30.00)
Comorbidity					
Diabetes	34,712 (12.45)	52,356 (12.13)	60,941 (11.93)	68,939 (11.84)	74,509 (11.78)
Hypertension	116,111 (41.64)	176,722 (40.95)	207,586 (40.63)	236,263 (40.57)	256,220 (40.49)
Cancer	5354 (1.92)	7613 (1.76)	8744 (1.71)	9776 (1.68)	10,371 (1.64)
Acute cardiovascular events					
Stroke	4392 (1.58)	9460 (2.19)	15,250 (2.98)	21,736 (3.73)	26,521 (4.19)
Acute myocardial infarction	203 (0.07)	509 (0.12)	934 (0.18)	1453 (0.25)	1872 (0.30)
Heart failure	3820 (1.37)	7346 (1.70)	11,847 (2.32)	17,477 (3.00)	22,010 (3.48)
Number of comorbidities					
0	147,529 (52.91)	229,721 (53.23)	270,608 (52.96)	305,562 (52.46)	330,868 (52.29)
1	100,059 (35.89)	153,591 (35.59)	181,406 (35.50)	206,510 (35.46)	223,030 (35.25)
2	29,240 (10.49)	44,616 (10.34)	53,458 (10.46)	62,647 (10.76)	69,077 (10.92)
≥3	1986 (0.71)	3650 (0.85)	5508 (1.08)	7705 (1.32)	9776 (1.54)
Vaccination history					
Received influenza vaccine during last influenza season	7741 (2.48)	13,360 (3.10)	27,139 (5.31)	143,778 (24.69)	232,405 (36.73)
Received COVID-19 vaccine before					
0 doses	0 (0.00)	0 (0.00)	0 (0.00)	366,637 (62.95)	39,754 (6.28)
1 dose	0 (0.00)	0 (0.00)	0 (0.00)	27,136 (4.66)	45,362 (7.17)
2 doses	0 (0.00)	0 (0.00)	0 (0.00)	188,651 (32.39)	139,370 (22.03)
≥3 dose	0 (0.00)	0 (0.00)	0 (0.00)	0 (0.00)	408,265 (64.52)
Received PPSV23 before					
0 doses	276,827 (99.29)	427,130 (98.97)	503,016 (98.44)	570,491 (97.95)	618,261 (97.71)
1 dose	1975 (0.71)	4413 (1.02)	7908 (1.55)	11,816 (2.03)	14,305 (2.26)
≥2 doses	12 (0.00)	35 (0.01)	56 (0.01)	117 (0.02)	185 (0.03)

^a^ Age groups were categorized by age as of 31 December 2022.

**Table 3:** Determinants associated with influenza vaccine uptake among the elderly with CLRDs.

Variable	aOR (95% CI)				
	2019–23	2019/20	2020/21	2021/22	2022/23
COVID-19 pandemic					
Before	Reference				
2020/21 season	12.71 (12.51–12.91)				
2021/22 season	7.31 (7.19–7.42)				
2022/23 season	3.98 (3.90–4.06)				
Demographic characteristics					
Gender					
Male	Reference	Reference	Reference	Reference	Reference
Female	1.15 (1.14–1.16)	1.34 (1.30–1.38)	1.10 (1.08–1.12)	1.16 (1.15–1.18)	1.13 (1.11–1.14)
Age groups (years) ^a^					
60–64	Reference	Reference	Reference	Reference	Reference
65–69	1.21 (1.19–1.23)	1.30 (1.24–1.37)	1.16 (1.12–1.20)	1.83 (1.75–1.90)	6.74 (6.13–7.42)
70–79	1.52 (1.49–1.55)	1.66 (1.59–1.74)	1.67 (1.61–1.74)	2.46 (2.35–2.56)	8.39 (7.62–9.24)
≥80	1.04 (1.01–1.06)	1.62 (1.54–1.71)	1.45 (1.39–1.52)	1.15 (1.10–1.21)	6.25 (5.67–6.89)
Region					
Rural	Reference	Reference	Reference	Reference	Reference
Urban	1.16 (1.16–1.17)	1.28 (1.24–1.32)	1.14 (1.12–1.16)	1.00 (0.99–1.02)	1.32 (1.31–1.34)
Immigration status					
Migrant	Reference	Reference	Reference	Reference	Reference
Resident	0.48 (0.47–0.49)	1.41 (1.29–1.54)	0.64 (0.61–0.67)	0.54 (0.51–0.56)	1.77 (1.61–1.94)
Eligible for free influenza vaccination	10.49 (10.31–10.67)	N/A	14.60 (14.10–15.12)	8.47 (8.16–8.80)	1.57 (1.42–1.73)
Diagnosed with ILI last influenza season	1.18 (1.17–1.19)	1.66 (1.59–1.72)	1.20 (1.18–1.21)	1.15 (1.14–1.17)	1.13 (1.12–1.15)
Comorbidity					
Diabetes	1.23 (1.22–1.25)	1.35 (1.30–1.41)	1.32 (1.29–1.35)	1.22 (1.20–1.25)	1.15 (1.13–1.18)
Hypertension	1.32 (1.31–1.33)	1.23 (1.19–1.27)	1.41 (1.39–1.43)	1.39 (1.37–1.40)	1.25 (1.23–1.27)
Cancer	1.19 (1.16–1.22)	1.15 (1.04–1.27)	1.42 (1.34–1.50)	1.07 (1.02–1.12)	1.16 (1.10–1.21)
Acute cardiovascular events					
Stroke	0.82 (0.80–0.83)	0.96 (0.87–1.06)	0.86 (0.82–0.89)	0.79 (0.77–0.82)	0.80 (0.78–0.83)
Acute myocardial infarction	0.88 (0.82–0.94)	1.31 (0.89–1.92)	0.77 (0.66–0.91)	0.83 (0.73–0.93)	0.94 (0.84–1.04)
Heart failure	0.84 (0.82–0.86)	1.40 (1.27–1.54)	0.92 (0.88–0.96)	0.76 (0.73–0.79)	0.82 (0.79–0.85)
Number of comorbidities					
0	Reference	Reference	Reference	Reference	Reference
1	1.28 (1.27–1.29)	1.22 (1.18–1.26)	1.36 (1.34–1.39)	1.33 (1.31–1.35)	1.22 (1.21–1.24)
2	1.33 (1.32–1.35)	1.46 (1.39–1.53)	1.50 (1.46–1.54)	1.34 (1.31–1.37)	1.21 (1.19–1.24)
≥3	1.15 (1.12–1.19)	1.55 (1.36–1.77)	1.37 (1.29–1.46)	1.11 (1.05–1.17)	1.04 (0.99–1.09)
Vaccination history					
Received influenza vaccine last influenza season	5.95 (5.89–6.00)	39.41 (37.81–41.08)	15.63 (15.08–16.20)	4.28 (4.22–4.35)	5.98 (5.90–6.06)
Received COVID-19 vaccine before					
0 doses	Reference	N/A	N/A	Reference	Reference
1 dose	1.12 (1.10–1.14)	N/A	N/A	1.38 (1.34–1.42)	0.68 (0.66–0.71)
2 doses	1.82 (1.80–1.84)	N/A	N/A	1.91 (1.88–1.93)	1.17 (1.14–1.20)
≥3 doses	2.93 (2.88–2.98)	N/A	N/A	N/A	2.03 (1.98–2.08)
Received PPSV23 before					
0 doses	Reference	Reference	Reference	Reference	Reference
1 dose	3.37 (3.27–3.46)	3.55 (3.25–3.88)	1.88 (1.77–2.01)	3.05 (2.90–3.20)	2.77 (2.64–2.90)
≥2 doses	4.23 (3.16–5.66)	2.15 (0.90–5.15)	6.03 (2.52–14.45)	3.64 (2.18–6.07)	2.82 (1.86–4.27)

Abbreviations: aOR: adjusted odds ratio; CI: confidence interval; N/A: not available. ^a^ Age groups were categorized by age as of 31 December 2022.

## Data Availability

The data that support the findings of this study are available from the Ningbo Health Information Center but restrictions apply to the availability of these data, which were used under license for the current study and are not publicly available. The data are, however, available from the authors upon reasonable request and with permission from the Ningbo Health Information Center.

## Supplementary Materials

The following supporting information can be downloaded at: https://www.mdpi.com/article/10.3390/vaccines13070705/s1, Supplementary Table S1: Diseases defined by diagnosis codes according to the International Classification of Diseases, Tenth Revision (ICD-10). Supplementary Table S2: The influenza vaccination coverage across different subgroups during 2018/19 to 2022/23 influenza seasons.

## Abbreviations

CLRD	Chronic lower respiratory disease
NRHIP	Ningbo Regional Health Information Platform
ZJIIMS	Zhejiang Immunization Information Management System
ILI	Influenza-like illness
