# Supplementary material for: Influenza Vaccination Coverage Among Elderly Patients with Chronic Lung Respiratory Disease in Ningbo, China: Impact of Free Vaccination Policies and the COVID-19 Pandemic
Source: Vaccines (Basel). 2025 Jun 29;13(7):705. doi: 10.3390/vaccines13070705 (PMC12299750; doi:10.3390/vaccines13070705)
Supplement: Supplementary file 1 [file vaccines-13-00705-s001.zip › vaccines-3692362-supplementary.pdf]

Supplementary Table S1 Diseases defined by diagnosis codes according to the International Classification of Diseases, Tenth Revision (ICD-10).

Diseases	ICD-10
**Influenza-like illness (ILI)**	
Cold	J00
Sinusitis	J01, J32
Pharyngitis	J02
Laryngitis, tracheitis or laryngotracheitis	J04
Upper respiratory tract infections	J06.8 or J06.9
Influenza	J09-J11
Pneumonia	J12-J18
Acute bronchitis, bronchiolitis (not specifically acute or chronic), obstructive bronchitis	J20, J40, J44.8
Fine bronchitis, capillary bronchitis	J21
Acute lower respiratory tract infections, nonspecific	J22
Chronic obstructive pulmonary disease combined with acute lower respiratory tract infection	J44.0
Chronic obstructive pulmonary disease with acute exacerbation	J44.1
Cough	R05
Pleurisy	R09.1
**Diabetes**	E10-E14
**Hypertension**	I10-I15
**Tumour**	C00-C97
**Acute cardiovascular events**	
**Stroke**	I60-I61, I63-I64
**Acute myocardial infarction**	I21, I22
**Heart failure**	I50

Supplementary Table S2 The influenza vaccination coverage across different subgroups during 2018/19 to 2022/23 influenza seasons.

Variables	Pre-COVID-19				During COVID-19					
	2018/19		2019/20		2020/21		2021/22		2022/23	
	N(%)	95%CI	N(%)	95%CI	N(%)	95%CI	N(%)	95%CI	N(%)	95%CI
Total	10015(3.59)	3.52-3.66	24253(5.62)	2.56-5.69	128785(25.20)	25.08-25.32	215914(37.07)	36.95-37.20	274131(43.32)	43.20-43.45
**Demographic characteristic**										
Gender										
Male	4515(3.29)	3.20-3.39	10314(4.87)	4.78-4.96	63269(25.23)	25.06-25.40	104371(36.45)	36.27-36.62	132838(42.79)	42.62-42.96
Female	5500(3.88)	3.78-3.98	13939(6.34)	6.24-6.44	65516(25.18)	25.02-25.35	111543(37.68)	37.50-37.85	141293(43.84)	43.67-44.01
Age groups (Years)[a]										
60-64	1241(2.17)	2.05-2.29	3443(3.71)	3.59-3.84	6233(5.57)	5.43-5.70	6940(5.38)	5.25-5.50	9516(6.61)	6.48-6.74
65-69	2290(3.12)	3.00-3.25	5817(5.03)	4.91-5.16	9331(6.77)	6.64-6.90	52427(33.21)	32.98-33.44	85099(49.05)	48.81-49.28
70-79	4191(4.13)	4.00-4.25	10363(6.66)	6.54-6.79	75307(41.10)	40.87-41.32	120463(57.62)	57.41-57.83	139898(61.83)	61.63-62.03
≥80	2293(4.92)	4.73-5.12	4630(6.84)	6.65-7.03	37914(48.68)	48.33-49.03	36084(41.77)	41.44-42.10	39618(44.52)	44.19-44.84
Region										
Rural	2930(2.71)	2.62-2.81	8713(4.60)	4.51-4.69	55313(24.00)	23.83-24.18	98755(37.14)	36.95-37.32	119434(41.25)	41.07-41.43
Urban	7085(4.15)	4.05-4.24	15540(6.42)	6.32-6.52	73472(26.19)	26.03-26.35	117159(37.02)	36.85-37.19	154697(45.07)	44.91-45.24
Immigration status										
Migrant	320(2.72)	2.44-3.03	722(3.78)	3.52-4.06	2239(9.43)	9.07-9.81	3721(13.19)	12.80-13.59	5996(18.09)	17.68-18.51
Resident	9695(3.63)	3.56-3.70	23531(5.70)	5.63-5.78	126546(25.97)	25.85-26.10	212193(38.29)	38.16-38.42	268135(44.72)	44.59-44.84
Eligible for free influenza vaccination	N/A	N/A	N/A	N/A	108126(51.73)	51.52-51.95	203267(50.33)	50.18-50.49	259135(55.30)	55.16-55.44
Diagnosed with ILI last influenza season	8694(3.83)	3.75-3.91	19987(6.32)	6.23-6.40	70552(26.84)	26.68-27.01	92539(39.57)	39.38-39.77	85337(44.96)	44.74-45.19
**Comorbidities**										
Diabetes	1800(5.19)	4.96-5.42	4268(8.15)	7.92-8.39	20075(32.94)	32.57-33.32	31298(45.40)	45.03-45.77	37811(50.75)	50.39-51.11
Hypertension	4761(4.10)	3.99-4.22	11779(6.67)	6.55-6.78	67071(32.31)	32.11-32.51	108585(45.96)	45.76-46.16	132361(51.66)	51.47-51.85
Tumour	282(5.27)	4.70-5.90	589(7.74)	7.16-8.36	3065(35.05)	34.06-36.06	4201(42.97)	41.99-43.96	5146(49.62)	48.66-50.58
Acute cardiovascular events										
Stroke	182(4.14)	3.59-4.78	599(6.33)	5.86-6.84	5409(35.47)	34.71-36.23	8587(39.51)	38.86-40.16	11217(42.29)	41.70-42.89
Acute myocardial infarction	12(5.91)	3.39-10.12	39(7.66)	5.65-10.32	274(29.34)	26.50-32.34	512(35.24)	32.82-37.73	766(40.92)	38.71-43.16
Heart failure	259(6.78)	6.03-7.62	708(9.64)	8.98-10.33	4991(42.13)	41.24-43.02	6963(39.84)	39.12-40.57	9270(42.12)	41.47-42.77

Number of comorbidities										
0	4535(3.07)	2.99-3.16	10671(4.65)	4.56-4.73	52008(19.22)	19.07-19.37	92554(30.29)	30.13-30.45	122800(37.11)	36.95-37.28
1	3794(3.79)	3.68-3.91	9549(6.22)	6.10-6.34	55232(30.45)	30.24-30.66	90470(43.81)	43.60-44.02	111237(49.88)	49.67-50.08
2	1563(5.35)	5.09-5.61	3685(8.26)	8.01-8.52	19183(35.88)	35.48-36.29	29310(46.79)	46.40-47.18	35391(51.23)	50.86-51.61
≥3	123(6.19)	5.21-7.34	348(9.53)	8.62-10.53	2362(42.88)	41.58-44.19	3580(46.46)	45.35-47.58	4703(48.11)	47.12-49.10
Received influenza vaccine last influenza season	3637(46.98)	45.87-48.10	8817(66.00)	65.19-66.79	19376(71.40)	70.85-71.93	99807(69.42)	69.18-69.66	177615(76.42)	76.25-76.60
Received COVID-19 vaccine before										
0 dose	N/A	N/A	N/A	N/A	N/A	N/A	124345(33.92)	33.76-34.07	16264(40.91)	40.43-41.40
1 dose	N/A	N/A	N/A	N/A	N/A	N/A	9997(36.84)	36.27-37.42	12611(27.80)	27.39-28.21
2 doses	N/A	N/A	N/A	N/A	N/A	N/A	81572(43.24)	43.02-43.46	54182(38.88)	38.62-39.13
≥3 doses	N/A	N/A	N/A	N/A	N/A	N/A	0(0.00)	N/A	191074(46.80)	46.65-46.95
Received PPSV23 before										
0 dose	9054(3.27)	3.21-3.34	21790(5.10)	5.04-5.17	123537(24.56)	24.44-24.68	207360(36.35)	36.22-36.47	263397(42.60)	42.48-42.73
1 dose	953(48.25)	46.05-50.46	2442(55.34)	53.87-56.80	5203(65.79)	64.74-66.83	8462(71.61)	70.79-72.42	10586(74.00)	73.28-74.71
≥2 doses	8(66.67)	37.59-86.91	21(60.00)	43.27-74.68	45(80.36)	67.91-88.78	92(78.63)	70.28-85.13	148(80.00)	73.61-85.15

Abbreviations: CI: Confidence Interval; N/A: Not available

[a] Age groups were categorized by age of December 31, 2022.
